# Development and validation of palmitoylation-related genes characteristics for prediction of prognosis and immune landscape in endometrial cancer

**DOI:** 10.1186/s41065-026-00699-2

**Published:** 2026-06-22

**Authors:** Chuchu Zhao, Guiying Zhang

**Affiliations:** Department of Obstetrics and Gynecology, Lishui Central Hospital, No. 289, Kuocang Road, Liandu District, Lishui, 323000 Zhejiang China

**Keywords:** Endometrial cancer, Palmitoylation, Prognostic model, Immune characteristic, Molecular subtype

## Abstract

**Background:**

Endometrial cancer (EC) is the most common cancer of the female reproductive system. Palmitoylation dysregulation is linked to various diseases. However, there is still insufficient research on its use as a prognostic marker for EC.

**Methods:**

This research utilized transcriptomic data and clinical characteristics from EC patients sourced from the TCGA database. Key palmitoylation-related genes (PRGs) were identified through a series of bioinformatics analyses, followed by the construction of a prognostic model. The mRNA expression levels of characteristic genes in EC cells and clinical samples were investigated through reverse transcription quantitative polymerase chain reaction (RT-qPCR). Immune infiltration analysis was performed using ssGSEA and CIBERSORT. The molecular subtypes of EC were identified by consensus clustering.

**Results:**

This study identified six PRGs, and the results of bioinformatic analysis of their mRNA expression patterns were consistent with experimental validation. A risk stratification model constructed based on these six PRGs was able to significantly distinguish between high-risk and low-risk patients in terms of survival prognosis, with patients in the low-risk group having a better prognosis than those in the high-risk group. Furthermore, immune characteristic heterogeneity was observed among different risk subgroups. Through consensus clustering analysis, EC samples were classified into two molecular subtypes with significant differences.

**Conclusion:**

This study constructed and validated a prognostic risk model for EC based on PRGs, which demonstrated potential for stratifying patient survival rates and characterizing immune microenvironment features in a retrospective dataset.

**Supplementary Information:**

The online version contains supplementary material available at https://doi.org/10.1186/s41065-026-00699-2.

## Highlights

Six PRGs (MOGAT3, PLA2G10, PLA2G2F, LCK, PLA2G2A, and ATP8A2) were identified and a prognostic risk model for EC with robust predictive power was constructed.

Two PRG-related EC molecular subtypes were identified, revealing marked differences in their immunological profiles.

Using the median risk score as a cutoff, patients were stratified into high- and low-risk groups. The low-risk group showed significantly better prognosis, and marked differences in immune characteristics were observed between the two risk groups.

**Supplementary Information:**

The online version contains supplementary material available at https://doi.org/10.1186/s41065-026-00699-2.

## Introduction

Endometrial cancer (EC) is a type of malignancy that originates from the endometrial epithelium and is currently experiencing a notable increase in incidence globally [1]. EC is mainly classified into endometrioid adenocarcinoma (type I) and estrogen-independent EC (type II) based on clinical and histological features [2]. Type I tumors are mainly endometrioid adenocarcinomas, which can be affected by an imbalance of luteinizing hormone and estrogen exposure or even obesity [3]. Type II tumors mainly include plasma carcinomas, clear cell carcinoma, undifferentiated carcinoma and carcinosarcoma, which have highly aggressive biological behaviors and poor prognosis [4]. Early-stage EC usually includes total hysterectomy and bilateral salpingo-oophorectomy, whereas in the case of advanced or recurrent EC, surgery is still essential but must be supplemented with systemic therapy such as chemotherapy, immunotherapy, targeted therapy and endocrine therapy [5]. Current diagnostic and therapeutic strategies for EC, include imaging techniques, pathologic diagnosis, surgical intervention, and chemotherapy, but these often impede late diagnosis and heterogeneous treatment response [6]. Against the backdrop of rapid advancements in precision medicine, identifying novel biomarkers for EC prognosis assessment and personalized treatment represents an urgent priority in this field.

Palmoylation as one of the major forms of post-translational modification of proteins [7]. By controlling the subcellular localization of tumor-associated proteins, maintaining their stability, and regulating their biological activity, it can intervene in the operation of carcinogenic signaling networks, thereby playing a key regulatory role in tumor initiation and progression [8, 9]. For example, palmitoylation of Neurotensin Receptor 1 (NTSR-1) at Cys381 and Cys383 sites in breast cancer inhibits ERK1/2 activation, thereby promoting serum deprivation-induced apoptosis [10]. In primary prostate cancer, mutations in the palmitoylation site lead to Src and Fyn kinase activity reduction, which impairs the clone formation ability of tumor cells [11]. These studies indicate that palmitoylation dynamically modulates the functional activity of oncogenes and tumor suppressors, exhibiting distinct expression patterns across different cancer types, suggesting its significant value as a potential therapeutic target [12].

In recent years, with the rapid development of biological databases and the increasing availability of public data, researchers now have access to extensive sample information covering multiple cancer types and their corresponding normal tissues. This enables large-scale, cross-cancer bioinformatics analyses and enhances the representativeness and broad applicability of related research findings [13]. Based on the above background, to explore the possible involvement of palmitoylation-related genes (PRGs) in EC, this study screened characteristic genes based on transcriptome data from EC patients in the TCGA database and developed a novel molecular subtyping approach to reveal immune infiltration features among different subtypes. This study has preliminarily identified potential prognostic biomarkers in EC, providing a reference for subsequent exploration of personalized treatment strategies.

## Materials and methods

### Data acquisition and preprocessing

We obtained mRNA expression profiles (normal: 35, tumor: 554), somatic mutations, and clinical data from the EC dataset in the TCGA database (TCGA-UCEC). After excluding samples without survival data and duplicates, the EC dataset was randomly divided into a training set (60%) and a validation set (40%). The CPTAC database was used to acquire the EC dataset (normal: 0, tumor: 123) as an independent external validation set. Additionally, we retrieved genes with the keyword “palmitoylation” from GeneCards, setting the relevance score > 5 and classifying them as protein-coding genes, ultimately identifying 317 PRGs.

### Differential analysis and enrichment analysis

In this study, based on the TCGA-UCEC dataset, we employed the calcNormFactors function from the edgeR package and further standardized the data using the TMM method. Subsequently, based on the normalized expression matrix, we conducted differential expression analysis, screening for genes with |logFC| > 1 and FDR < 0.05. Based on the “c2.cp.kegg.v7.5.symbols.gmt gene set”, the clusterProfiler package was used to perform GSEA on the characteristic genes (adjusted p-value < 0.05, |NES| > 1). For each signature gene, the five pathways with the most significant adjusted p-value were selected for display.

### Screening of characteristic genes and construction of prognostic model

Based on the differentially expressed PRGs identified previously, this study further employed a stepwise screening strategy to identify characteristic genes. First, univariate Cox regression analysis was performed to preliminarily screen for genes significantly associated with prognosis. Subsequently, the Random Forest algorithm was used to assess the importance of the preliminarily screened genes, with the model parameters set as follows: the number of decision trees was 100 (ntree = 100), the number of variables considered at each split was 10 (nsplit = 10), and Out-Of-Bag (OOB) error estimation was adopted to evaluate the importance score of each variable (importance > 0.02). Finally, the key genes selected by Random Forest were incorporated into a multivariate Cox regression model to further filter the characteristic genes. The risk score was then determined according to the formula presented below:

$$\begin{aligned}Risk\:score=&\sum\:({Gene\:expression}_{i}\\&\times{Coefficient}_{i})\end{aligned}$$

To evaluate the model performance, this study was validated on the TCGA training set, TCGA validation set, CPTAC validation set, and complete TCGA-UCEC dataset. By plotting receiver operating characteristic (ROC) curve and calculating the corresponding AUC, the model’s predictive accuracy for patient outcomes at different time points was quantified.

### Construction of competitive endogenous RNA network

Using the multiMiR package, we obtained miRNAs interacting with characteristic genes from the TargetScan database. We downloaded lncRNA-miRNA interaction data from the ENCORI database, and screened out lncRNAs with clipExpNum > 10. Visualization was performed using Cytoscape (3.10.2).

### Nomogram based on clinical information and risk score

The training cohorts incorporated the clinical characteristics and risk scores for prognosis assessment. The independence of the prognostic model was established using both univariate and multivariate regression analyses. The clinical utility of the model was evaluated through decision curve analysis (DCA), and the nomogram was assessed using calibration curves.

### Immune infiltration analysis

This study applied the ssGSEA and CIBERSORT algorithms to evaluate immune cell infiltration between high‑ and low‑risk groups. ESTIMATE was used to calculate ESTIMATE scores, immune scores, stromal scores, and tumor purity across different risk subgroups. The immunophenoscore (IPS) for EC was obtained from the TCIA database to predict the response of high‑ and low‑risk patients to anti‑CTLA‑4 and anti‑PD‑1 therapies. Furthermore, the TIDE algorithm assessed immune evasion potential, and immune checkpoint expression levels were compared among the risk groups.

### Tumor mutational burden profiling

From the TCGA-UCEC mutation data, we identified and statistically analyzed the 20 most common recurring mutations separately for the high‑risk and low‑risk groups. Waterfall plots were produced via the GenVisR package, and the Wilcoxon test served to evaluate whether tumor mutational burden (TMB) scores differed significantly between the two risk groups.

### Assessment of the drug sensitivity

To discover potential therapeutic agents directed against the characteristic genes, the CellMiner database was screened for antitumor drugs showing strong associations with these genes. Subsequently, the pRRophetic package was applied to estimate the half maximal inhibitory concentration (IC_50_) values of multiple drugs in the high‑risk and low‑risk groups.

### Molecular subtyping

Consensus clustering was performed using the characteristic genes obtained from the prognostic model to define molecular subtypes of EC. The cumulative distribution function (CDF) and delta area curve were used to evaluate the stability of the resulting clusters.

### Reverse transcription quantitative polymerase chain reaction (RT-qPCR)

The human endometrial adenocarcinoma cell line HEC1A and the human endometrial epithelial cell line hEM3 used in this study were cultured in DMEM medium under strictly aseptic conditions. Five patients who were pathologically diagnosed with EC after surgery and underwent surgical resection at our hospital were enrolled in this study. The inclusion criteria were as follows: (1) pathologically confirmed EC; (2) no prior radiotherapy, chemotherapy, hormonal therapy, or targeted therapy before surgery; (3) no history of other malignancies; and (4) complete clinical and pathological data. The exclusion criteria were: (1) concurrent uterine fibroids, endometrial hyperplasia, or other gynecological malignancies; (2) concurrent severe dysfunction of major organs; (3) concurrent immune or coagulation dysfunction; and (4) receipt of neoadjuvant therapy before surgery. During surgery, tumor tissues and paired adjacent non‑tumor tissues were collected (a total of 5 pairs, i.e., 5 tumor tissues and 5 adjacent tissues). The tissue samples were snap‑frozen in liquid nitrogen and stored at − 80 °C. This study was approved by the hospital’s ethics committee, and all patients signed informed consent forms.

TRIzol reagent was used to extract total RNA from cells and tissues. Reverse transcription was then carried out with PrimeScript™ RT Master Mix to produce cDNA. A NanoDrop 2000 spectrophotometer was employed to assess the concentration and purity of both RNA and cDNA. RT-qPCR was conducted on a QuantStudio 5 real‑time PCR system, with primer sequences provided in Supplementary Table S1. GAPDH served as the internal control, and relative target gene expression was determined using the 2⁻ΔΔCt method.

### Statistical analysis

Utilizing R software (version 4.4.1), data were analyzed and visualized. The Wilcoxon test was used to assess differences between groups, and Pearson correlation coefficients were calculated to quantify relationships, with statistical significance defined as *p* < 0.05.

## Results

### Identification and functional enrichment of PRGs in EC

**Fig. 1:**
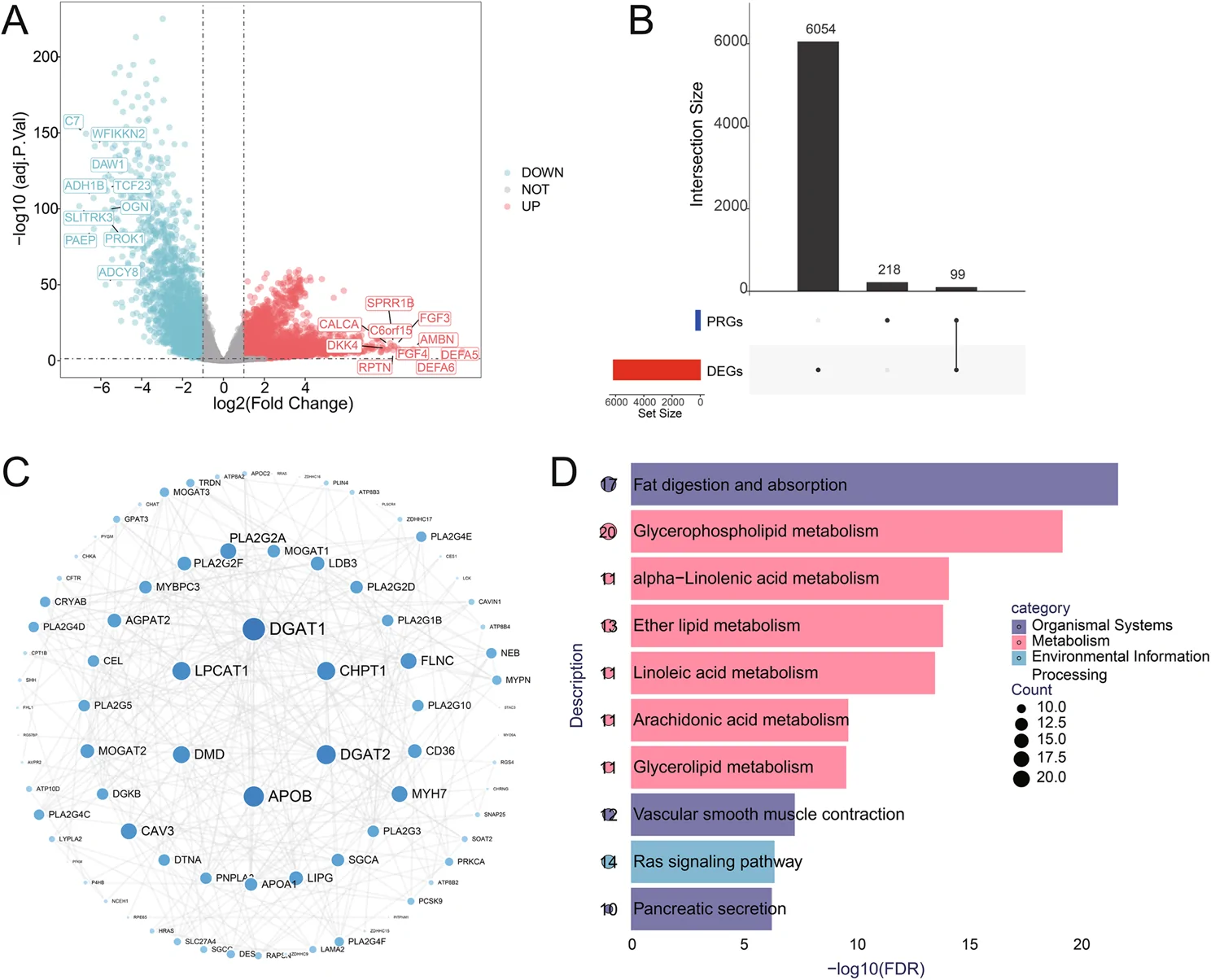
Identification and functional enrichment of PRGs in EC. **A** Volcano plot of DEGs (|logFC| > 1, FDR < 0.05). **B** Intersection analysis plot of DEGs and PRGs. **C** PPI network plot of difference PRGs. **D** KEGG enrichment analysis of differential PRGs

Differential analysis based on TCGA-UCEC data identified a total of 6153 significantly differentially expressed genes (DEGs) (Fig. 1A). After intersection analysis of DEGs with a collection of PRGs, 99 differentially expressed PRGs were screened out (Fig. 1B) and a PPI network was constructed (Fig. 1C). Subsequent KEGG enrichment analysis indicated (Fig. 1D) that differential PRGs were enriched in glycerophospholipid metabolism, ether lipid metabolism, and glycerolipid metabolism. Similar enrichment results were obtained by GO enrichment analysis (Supplementary Figure S1).

### Prognostic model development and validation

**Fig. 2:**
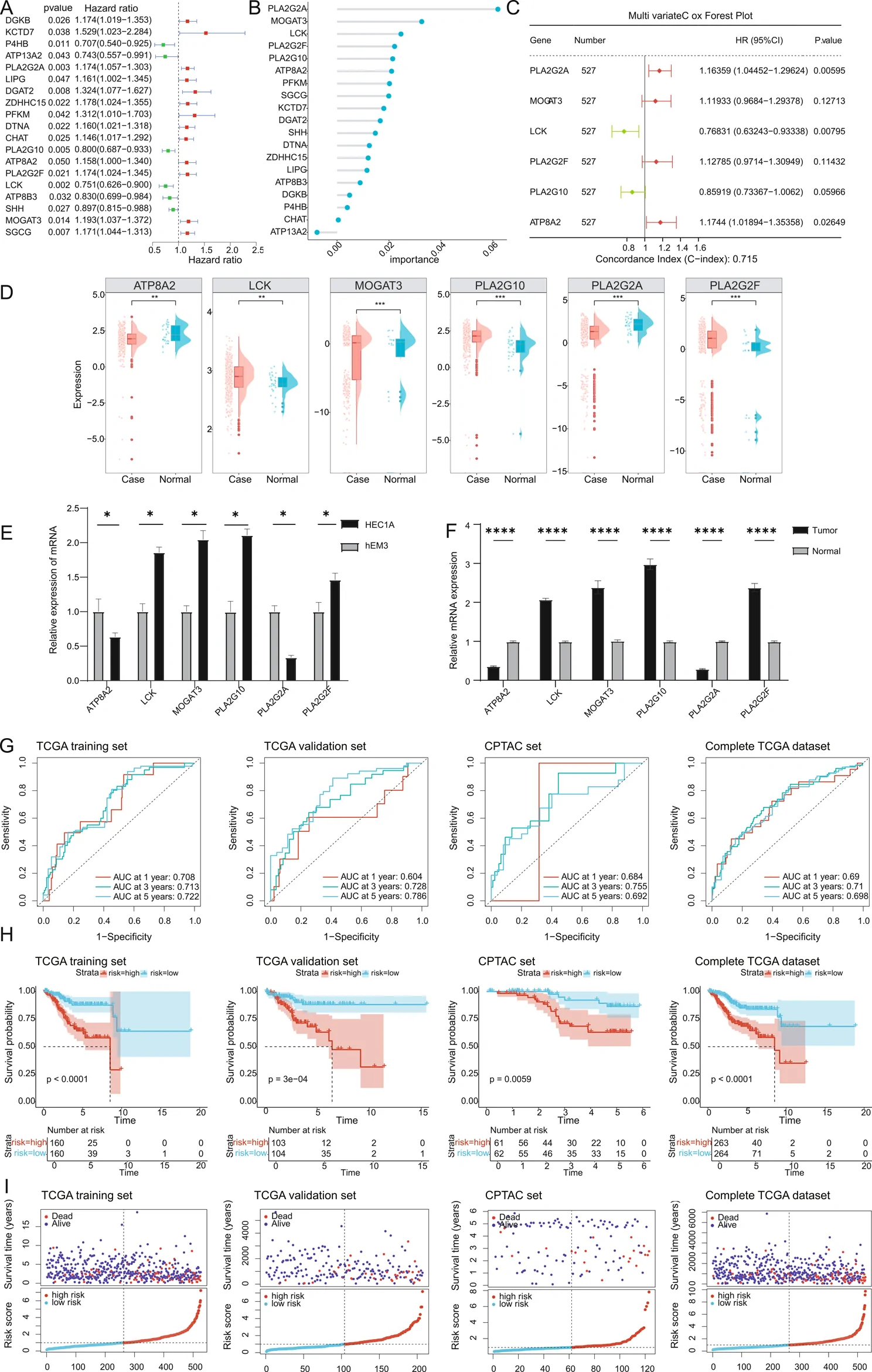
Prognostic model development and validation. **A** Univariate Cox regression for screening potential characteristic genes. **B** Random Forest analysis for identifying characteristic genes. **C** Multivariate Cox regression analysis for identifying characteristic genes. **D** Expression level differences of characteristic genes between case and normal groups. **E** Determine the mRNA expression levels of specific genes in human endometrial adenocarcinoma cells (HEC1A) and human endometrial epithelial cells (hEM3) using RT-qPCR. **F** Analyze the mRNA expression levels of specific genes in EC tissue (tumor) and adjacent non-cancerous tissues (normal) using RT-qPCR. **G**–**I** Evaluation of the prognostic model’s predictive performance and risk stratification in the TCGA training set, TCGA validation set, CPTAC dataset, and complete TCGA dataset: **G** ROC curves showing the predictive accuracy of the model; **H** K-M survival curves demonstrating the survival differences between high-risk and low-risk patients; **I** risk score distribution and survival status scatter plots illustrating patient risk stratification and survival outcomes. *: *p* < 0.05, **: *p* < 0.01, ***: *p* < 0.001

Univariate Cox regression (*p* < 0.05) drawing on previously screened differential PRGs identified 19 candidate genes that were significantly associated with patient survival (Fig. 2A). Using Random Forest analysis (importance > 0.02), 7 candidate genes were obtained (Fig. 2B). Further multivariate Cox regression analysis was applied to obtain 6 characteristic genes and construct a prognostic model (Fig. 2C).

$$\begin{aligned}Riskscore=&0.152\times\mathrm{P}\mathrm{L}\mathrm{A}2\mathrm{G}2\mathrm{A}+0.113\\&\times\mathrm{M}\mathrm{O}\mathrm{G}\mathrm{A}\mathrm{T}3-0.264\times\mathrm{L}\mathrm{C}\mathrm{K}\\&+0.120\times\mathrm{P}\mathrm{L}\mathrm{A}2\mathrm{G}2\mathrm{F}-0.152\\&\times\mathrm{P}\mathrm{L}\mathrm{A}2\mathrm{G}10+0.161\times\mathrm{A}\mathrm{T}\mathrm{P}8\mathrm{A}2\end{aligned}$$

Subsequent differential analysis indicated that LCK, MOGAT3, PLA2G10, PLA2G2F were significantly highly expressed in EC, and ATP8A2, PLA2G2A were significantly low expressed in EC (Fig. 2D). RT-qPCR experiments further validated the aforementioned mRNA expression trends in cell lines and clinical samples, with results consistent with the bioinformatics analysis (Fig. 2E–F). The prognostic model demonstrated strong prediction power in both the training and validation cohorts, as evidenced by ROC curve analysis (Fig. 2G). K‑M survival analysis also showed that patients in the low‑risk group had a better prognosis than those in the high‑risk group (Fig. 2H). The risk score distributions and corresponding survival status plots provided clear evidence of outcome stratification for each cohort (Fig. 2I).

### Construction of nomogram

**Fig. 3:**
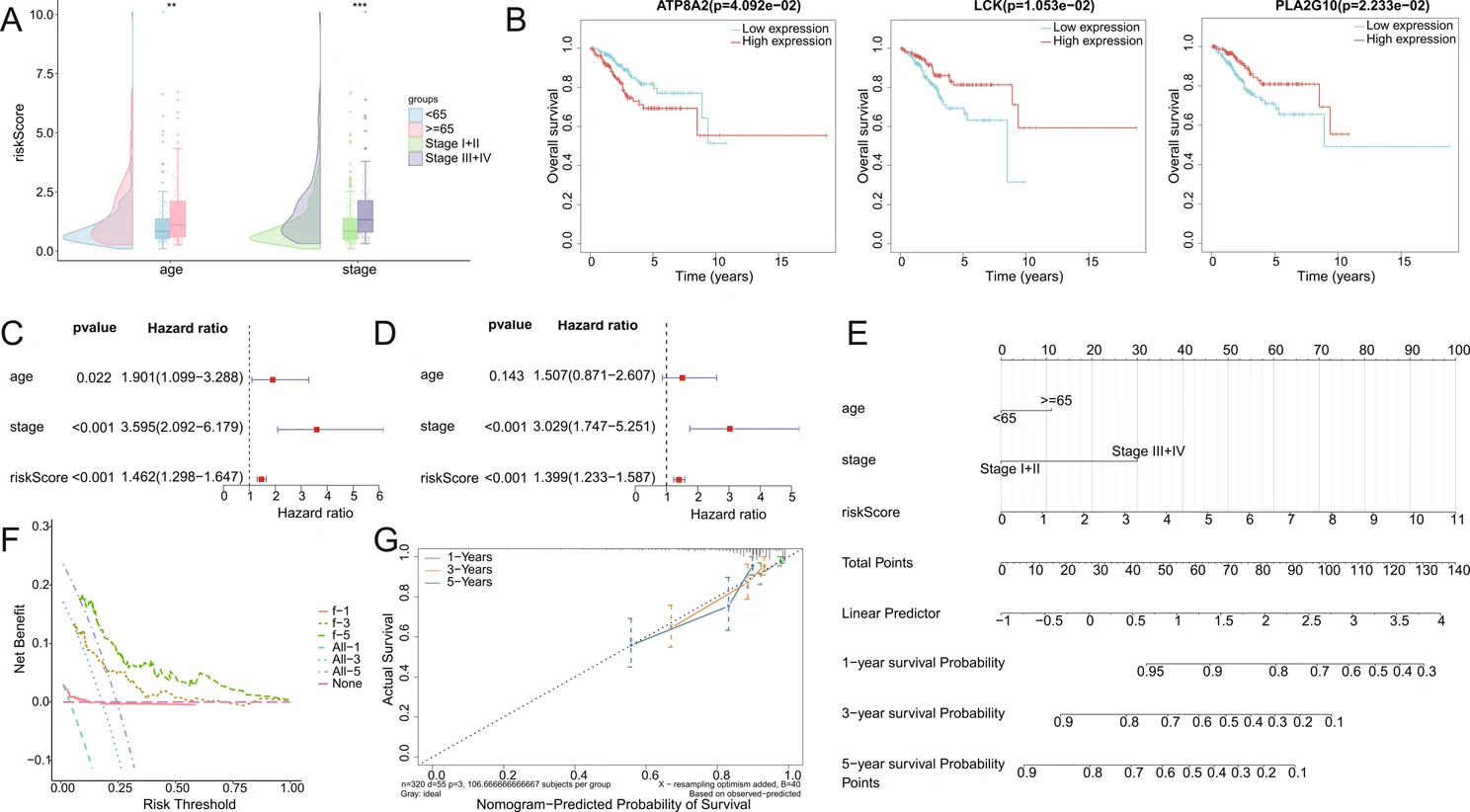
Construction of nomogram. **A** Distribution of riskscores for clinical characteristics. **B** K-M survival analysis of characteristic genes. **C** Univariate Cox regression analysis of prognostic factors. **D** Multivariate Cox regression analysis of prognostic factors. **E** Nomogram constructed by integrating clinical parameters and riskscores. **F** DCA curve analysis of the nomogram. **G** Calibration curve for the nomogram. **: *p* < 0.01, ***: *p* < 0.001

Patients aged 65 years or older were found to have significantly higher risk scores than younger patients, and those with stage III‑IV disease had markedly higher risk scores than stage I‑II patients (Fig. 3A). K‑M survival analysis revealed that low ATP8A2 expression predicted better overall survival, while high LCK and PLA2G10 expression were linked to improved survival compared with low expression (Fig. 3B). Both univariate and multivariate regression analyses, which included risk scores and clinical features, demonstrated that stage and risk score each serve as significant independent predictors of prognosis (Fig. 3C‑D). To predict individual survival probabilities, a nomogram integrating risk scores with key clinical variables was constructed (Fig. 3E). The model’s clinical applicability was evaluated using DCA and calibration curves, which confirmed its high predictive accuracy for 1‑, 3‑, and 5‑year survival (Fig. 3F‑G).

### Immune infiltration analysis

**Fig. 4:**
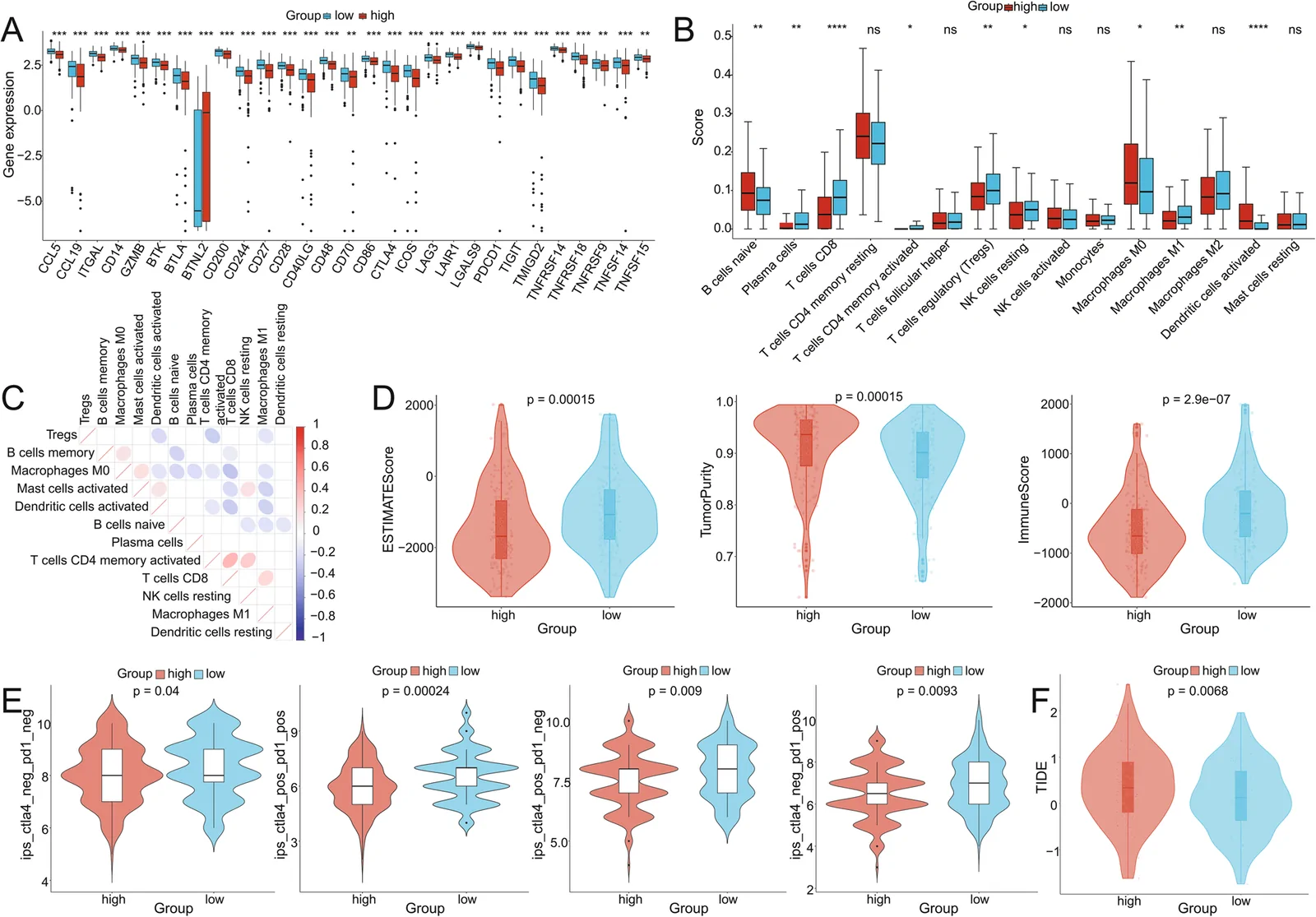
Immune infiltration analysis. **A** Immune checkpoint expression across risk groups. **B** Analysis of immune function and cellular infiltration scores in different risk groups using the CIBERSORT algorithm. **C** Correlation of differentially infiltrating immune cells. **D** Immune stromal characterization scores across risk groups. **E** IPS score violin plot. **F** TIDE score violin plot. ns: not significant, *: *p* < 0.05, **: *p* < 0.01, ***: *p* < 0.001, ****: *p* < 0.0001

The immune checkpoint expression profile (Fig. 4A) revealed that while BTNL2 showed upregulated expression in the high-risk group. Further CIBERSORT analysis of immune infiltration revealed that the high-risk group had higher levels of infiltration by B cells naive, Macrophages M0, and Dendritic cells activated. Conversely, Plasma cells, T cells CD8, T cells CD4 memory activated, Tregs, NK cells resting, and Macrophages M1 showed more significant infiltration in the low-risk group (Fig. 4B). Notably, T cells CD8 showed positive correlation with T cells CD4 memory activated but negative correlation with Macrophages M0 (Fig. 4C). ESTIMATE algorithm analysis revealed significant differences in tumor microenvironment (TME) composition among different risk groups. The low-risk group exhibited significantly elevated ESTIMATEScore and ImmuneScore, while TumorPurity was notably lower than the high-risk group (Fig. 4D). Subsequent analysis of immune phenotype scores across the two risk groups demonstrated that the low-risk group exhibited stronger immune responses against CTLA-4 and PD-1 compared to the high-risk group (Fig. 4E). TIDE analysis further indicated that the high-risk group showed significantly higher TIDE values than the low-risk group (Fig. 4F).

### GSEA based on characteristic genes

**Fig. 5:**
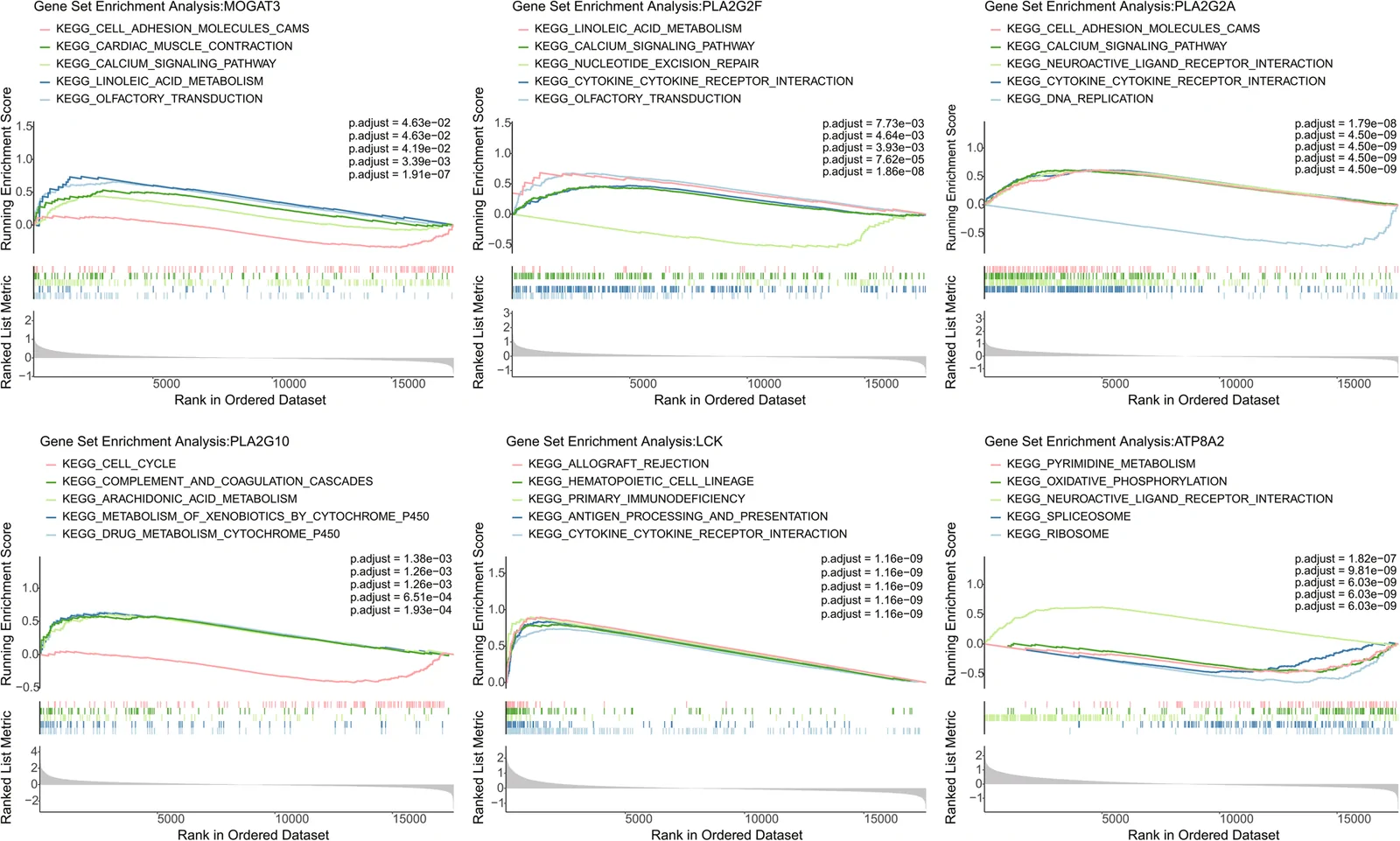
GSEA based on characteristic genes

To investigate the biological features of characteristic genes in EC, we performed GSEA. The results showed that MOGAT3, PLA2G2F and PLA2G2A were co-enriched in the calcium signaling pathway. MOGAT3 and PLA2G2A were co-enriched in the cell adhesion molecules cams signaling pathway. The cytokine-receptor interaction pathway showed significant enrichment in PLA2G2F, PLA2G2A, and LCK. Additionally, each gene exhibited specific biological pathways. MOGAT3 was enriched in linoleic acid metabolism and olfactory transduction pathways, PLA2G2F involved nucleotide excision repair, PLA2G2A was enriched in neuroactive ligand receptor interaction and DNA replication, while PLA2G10 primarily participated in cell cycle and complement coagulation cascades. The LCK is involved in immune activation (allograft rejection, antigen processing and presentation), while ATPRA2 focuses on oxidative phosphorylation, pyrimidine metabolism and ribosome (Fig. 5).

### Tumor mutation burden and drug sensitivity analysis

**Fig. 6:**
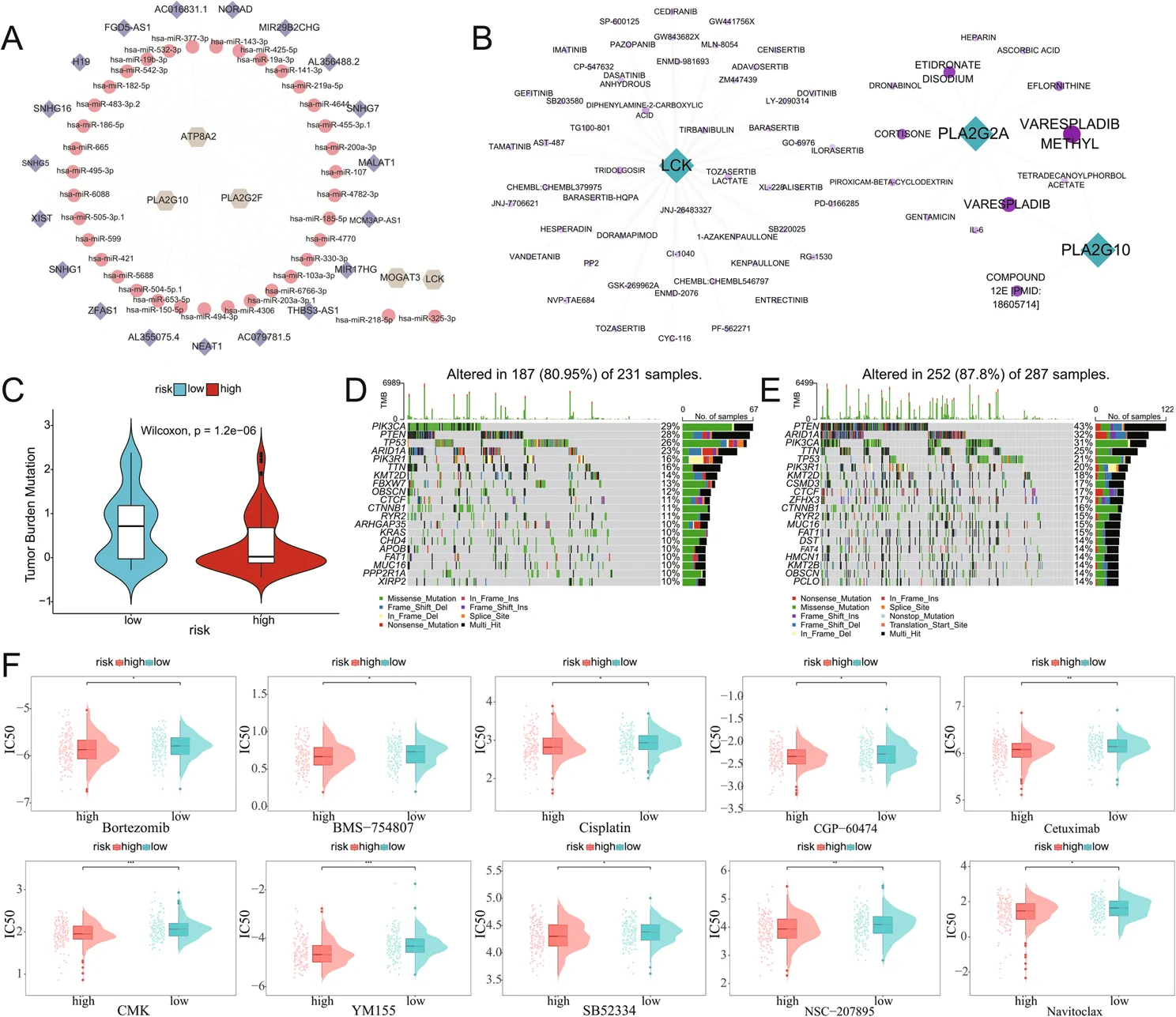
Tumor mutation burden and drug sensitivity analysis. **A** ceRNA regulatory network based on characteristic genes. **B** Prediction of small molecule drugs with potential interactions among characteristic genes. **C** Violin plot illustrating the differences in TMB between high risk and low risk patient groups. **D** TMB waterfall chart for high-risk patients. **E** TMB waterfall chart for low-risk patients. **F** Ten potential therapeutic agents predicted to exhibit higher sensitivity in high-risk patients. *: *p* < 0.05, **: *p* < 0.01, ***: *p* < 0.001

The construction of ceRNA network based on characteristic genes (Fig. 6A) revealed that MALAT1 regulates ATP8A2 by binding to hsa-miR-200a-3p, while also modulates PLA2G2F through interaction with hsa-miR-4644. Additionally, FGD5-AS1 regulates PLA2G10 via binding to hsa-miR-19b-3p. Subsequent, using DGIdb database to predict small molecule drug interactions with characteristic genes (Fig. 6B) indicated that varespladib may exert anticancer effects by targeting PLA2G2A and PLA2G10. TMB analysis based on risk stratification of patient mutation profiles showed significantly lower TMB levels in high-risk groups in comparison with low-risk groups (Fig. 6C), with lower PTEN mutation frequency observed in high-risk patients (28%) than in low-risk patients (43%) (Fig. 6D-E). Additionally, this study identified 10 potential therapeutic agents with heightened sensitivity in high-risk patients (Fig. 6F), which are: Bortezomib, BMS-754,807, Cisplatin, CGP-60,474, Cetuximab, CMK, YM155, SB52334, NSC-207,895 and Navitoclax. Using the CellMiner database, the association between characteristic gene expression and drug sensitivity was analyzed (Supplementary Fig. 2), in which the correlation coefficient between PLA2G10 and YM155 was 0.546 (*p* < 0.001), suggesting a potential correlation between the two.

### Molecular subtyping of EC

**Fig. 7:**
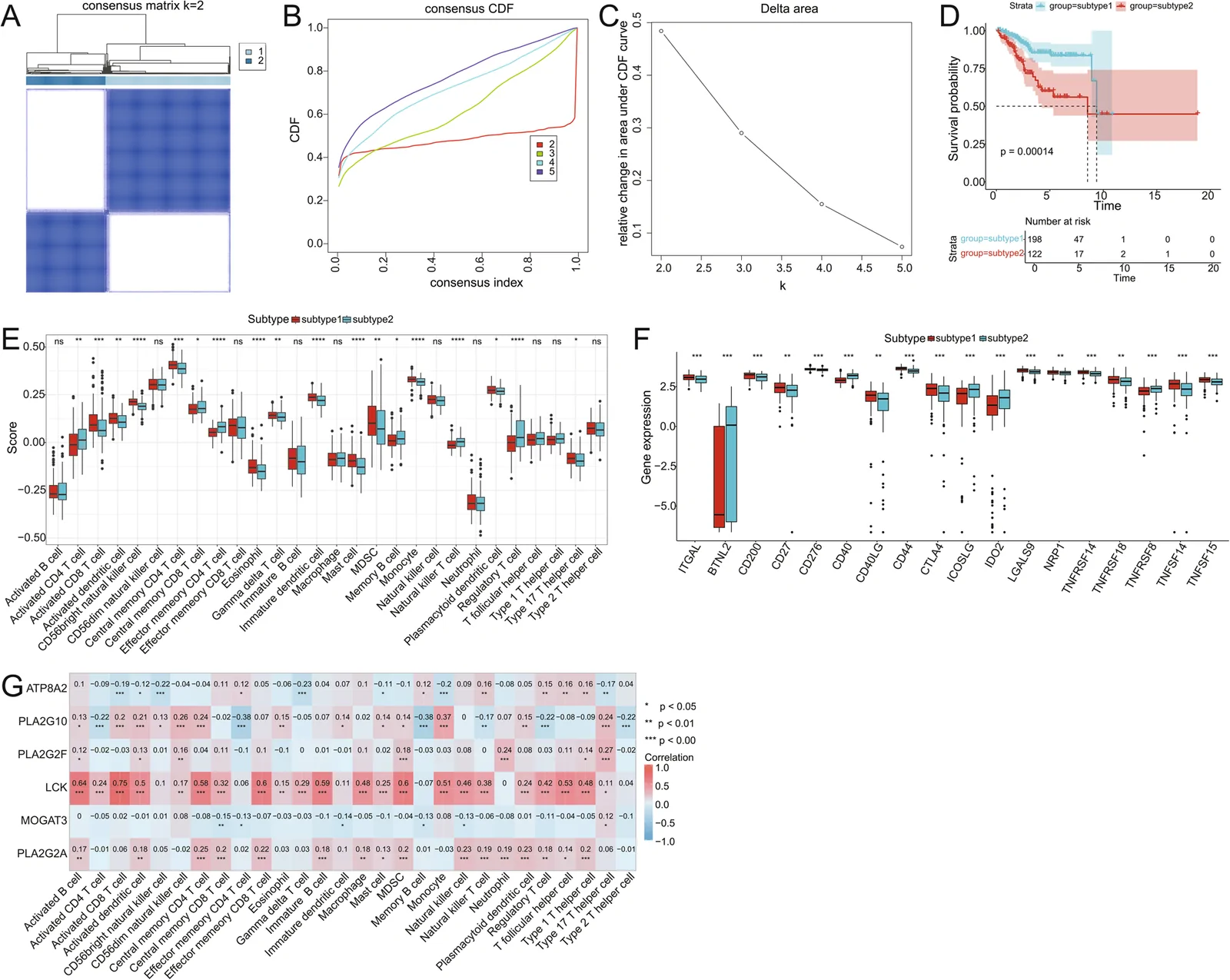
Molecular subtyping of EC. **A** Heatmap of consensus clustering (k = 2). **B** CDF for cluster stability assessment. **C** Delta area curve. **D** K-M survival analysis to evaluate survival in different patient subgroups. **E** ssGSEA immune infiltration analysis. **F** Analysis of immune checkpoint molecule expression level differences among subtypes. **G** Analysis of the correlation between characteristic genes and immune cells. ns: not significant, *: *p* < 0.05, **: *p* < 0.01, ***: *p* < 0.001, ****: *p* < 0.0001

Two molecular subtypes of TCGA-UCEC samples were established using the characteristic genes (Fig. 7A). The stability of this classification was well supported by CDF and delta area curves (Figs. 7B‑C). K‑M survival analysis demonstrated that patients in subtype 1 experienced markedly better survival than those in subtype 2 (Fig. 7D). ssGSEA analysis revealed pronounced differences between the two subtypes across 18 immune cell types (Fig. 7E). When immune checkpoint genes were compared between subtypes, all 18 showed significant differential expression (Fig. 7F). Additionally, correlation analysis linking characteristic genes to immune cell types found that LCK was significantly correlated with 22 specific immune cell populations (Fig. 7G).

## Discussion

EC is the most prevalent gynecological malignancy in high-income nations, experiencing a rising global incidence [14]. EC can be classified into four types based on molecular typing: POLE mutant type, mismatch repair deficiency type, p53 abnormal type, and no specific molecular characteristics type [15]. Palmitoylation regulates key properties in protein biology, including molecular stability, membrane binding, intermolecular interactions, and signal transduction, and thus plays an indispensable role in a wide range of physiological and pathological processes [16–18]. Based on this, this study focuses on PRGs and systematically analyzes the mechanisms related to EC.

In this study, we identified six characteristic genes (MOGAT3, PLA2G10, PLA2G2F, LCK, PLA2G2A, and ATP8A2) and performed cross-validation, which suggested potential functional links with certain pathogenic mechanisms of EC. Monoacylglycerol O-acyltransferase 3 (MOGAT3) catalyzes the acylation of monoacylglycerol (MAG) and diacylglycerol (DAG), thereby promoting DAG synthesis [19]. In metastatic colorectal cancer, MOGAT3 upregulation enhances DAG production, suppresses fatty acid oxidation, and facilitates DAG accumulation, this accumulated DAG activates the PKCα/CRAF/MEK/ERK signaling cascade, driving the development of acquired drug resistance [20]. Lymphocyte-specific protein tyrosine kinase (LCK), a member of the Src family of protein tyrosine kinases (PTKs), plays significant roles in cancer pathogenesis [21]. For instance, in cholangiocarcinoma, LCK expression correlates with tumor recurrence [22]. In endometrioid carcinoma, LCK is implicated in the upregulation of DNA repair genes (MLH1 and BRCA1) and contributes to cisplatin resistance in cancer stem cells (CSCs) [23]. In addition, LCK undergoes post-translational modification via palmitoylation, which has been identified as a key mechanism regulating its biological function, membrane affinity, and localization to sphingolipid-rich microdomains [24]. Phospholipase A2 group IIA (PLA2G2A), a member of the phospholipase A2 (PLA2) family, modulates CD8 + T cell function via the MAPK/Erk and NF-κB signaling pathways in pancreatical ductal adenocarcinoma (PDAC) [25]. Overexpression of phospholipase A2 group X (PLA2G10) is associated with poor T-cell infiltration in tumor tissues, in immunogenic mouse tumors induces T cell exclusion from the TME, conferring resistance to anti-PD-1 immunotherapy [26]. Our findings indicate that PLA2G10 mRNA levels were elevated in HEC1A cells, and the high-risk patient group may have a relatively lower response to anti-PD-1 therapy compared to the low-risk group. Whether PLA2G10 upregulation in EC similarly impairs T cell infiltration and leads to resistance against anti-PD-1 immunotherapy warrants further investigation. ATPase phospholipid transporter 8A2 (ATP8A2) actively transports phosphatidylserine and phosphatidylethanolamine from the outer leaflet to the inner leaflet of the cell membrane, thereby generating and maintaining phospholipid asymmetry [27]. This study observed that, in survival analysis, low expression of ATP8A2 was associated with a better survival prognosis; however, in multivariate risk models, it was assigned a positive coefficient. This seemingly contradictory result is similar to recent findings in gastric cancer [28], revealing the complexity of the role of genes in cancer prognosis. From a statistical perspective, univariate analysis estimates the marginal effect of ATP8A2 without adjusting for other covariates, whereas multivariate analysis assesses its net independent contribution after controlling for co-expressed genes or clinical factors. When the expression of ATP8A2 is negatively correlated with other prognostic genes, a “suppression effect” may occur, leading to a reversal in the sign of the coefficient [29, 30]. Based on these findings, we hypothesize that ATP8A2 may function as a context-dependent gene, with its risk effect potentially dominating in multivariate models. This suggests that when ATP8A2 is co-expressed with other specific genes (such as PLA2G10 in the model), they may synergistically promote tumor progression. Future studies will employ functional experiments to validate the specific mechanisms underlying its association with immune infiltration or chemotherapy resistance in specific tumor contexts, under conditions where ATP8A2 is regulated individually or in combination with other genes. Additionally, our research indicates that phospholipase A2 group IIF (PLA2G2F) may participate in nucleotide excision repair. In summary, these signature genes may be regulated directly or indirectly by palmitoylation, participating in the regulation of lipid metabolism and membrane function, thereby influencing the progression of EC.

Regulation of the immune microenvironment plays a significant role in EC [31]. Analysis of the ESTIMATE algorithm showed that the low-risk group had significantly higher ESTIMATEScore and ImmuneScore and lower TumorPurity, suggesting that their good prognosis is closely related to abundant tumor immune infiltration. Analysis of immune checkpoint expression further supported this conclusion, with only butyrophilin-like protein 2 (BTNL2) showing upregulated expression in the high-risk group. Research reports that BTNL2 promotes the production of interleukin 17 A (IL-17 A) by acting on γδ T cells in the TME, thereby recruiting myelioid-derived suppressor cells (MDSCs). It leads to a reduction in the infiltration of cytotoxic CD8 + T cells, thereby inhibiting anti-tumor immunity [32]. The expression of checkpoint molecules such as BTNL2 increased after anti-programmed death receptor 1 (PD-1) treatment, suggesting that BTNL2 may represent a new immune evasion mechanism [33]. In addition, in lung adenocarcinoma and colonic adenocarcinoma, low levels of BTNL2 expression are significantly associated with an increased survival period of patients [32]. In summary, BTNL2 may serve as a potential biomarker for the immunosuppressive microenvironment of EC; however, its mechanism of action requires further elucidation to advance the development of BTNL2-targeted antibody inhibitors against EC. TMB analysis revealed significantly lower TMB levels and a decreased PTEN mutation frequency in the high-risk group relative to the low-risk group. Consistent with previous studies, reduced PTEN mRNA expression in EC has been associated with poor prognosis [34]. PTEN plays an important role in tumor suppression by regulating PTEN/PI3K/AKT and FRAP/mTOR signaling pathways [35]. It is worth noting that the finding of lower TMB and lower PTEN mutation rates in the high-risk group, while interesting, is counterintuitive. We believe this phenomenon can be reasonably explained from the perspective of the traditional pathological classification of EC: EC is primarily divided into Type I (predominantly endometrioid tumors) and Type II (predominantly serous tumors). Among these, PTEN mutations are commonly observed in the early stages of Type I tumors [36], whereas TP53 mutations are more frequently seen in Type II tumors [37]. The results of this study show a higher frequency of TP53 mutations in the high-risk group (26% vs. 21%), suggesting that this group may be enriched with Type II tumor patients. Although these patients have relatively low TMB, their tumors are more aggressive and therefore still exhibit poor clinical outcomes [38, 39]. In addition, age may also play a role in shaping this mutation profile: previous studies have shown that PTEN mutations are relatively more common in younger EC patients, whereas older patients exhibit a higher frequency of TP53 mutations [40]. Considering the combined effects of age and EC subtypes on prognosis may provide some insight into why high-risk groups continue to experience aggressive clinical courses and poor outcomes, even when TMB is low.

KEGG enrichment analysis revealed that differentially expressed PRGs were significantly enriched in lipid metabolism pathways, consistent with previous reports and highlighting the close interaction mechanism between palmitoylation and lipid metabolism. Palmitylation is an important protein post-translational modification [41]. Lipid metabolism is responsible for the synthesis, breakdown and utilization of fatty acids [42]. The two interact with each other to regulate a variety of cellular processes, particularly in energy homeostasis and organelle function [43]. For example, artemisinin can covalently bind and inhibit ZDHHC palmitoyltransferase 6 (ZDHHC6), thereby blocking the palmitoylation of NRas proto-oncogene (NRas), altering its subcellular localization, and ultimately disrupting carcinogenic signaling pathways [44].

In summary, this study not only identified six PRGs with independent prognostic value but also revealed their potential associations with unique immune microenvironment characteristics, latent therapeutic sensitivity, and molecular subtyping, offering new insights into understanding the heterogeneity of EC. However, several limitations remain. First, in terms of mechanistic validation, the current conclusions are primarily derived from correlation analyses and feature selection using public databases, lacking direct experimental evidence. Specifically, no in vitro or in vivo functional experiments, such as gene knockout/overexpression or site-directed mutagenesis of palmitoylation sites for key PRGs, have been conducted. Therefore, the causal roles and specific molecular pathways of these genes in EC development, immune evasion, and tumor proliferation and invasion remain unclear. Second, regarding data sources, the analyses rely entirely on retrospective public datasets, with relatively limited sample sizes and insufficient diversity in patient clinical characteristics (e.g., ethnicity, treatment history, disease stage), which may affect the generalizability and extrapolation of the results. Although random grouping and external dataset validation were adopted, the model still lacks prospective, multicenter, and multi-ethnic independent cohorts for external validation, and its actual clinical application value needs further verification. Furthermore, the model construction process relies on heuristic parameter selection, and under limited sample sizes, the random forest model may introduce estimation bias in OOB error and instability in feature importance, making it impossible to fully exclude the risk of overfitting. Future research will aim to integrate multi-omics data (genomics, epigenomics, proteomics), conduct systematic gain- and loss-of-function experiments (e.g., in vivo tumor formation models, organoid drug sensitivity tests), and validate the model’s efficacy and generalizability in larger, multicenter cohorts, thereby providing a more robust basis for precise risk stratification and treatment strategy optimization in EC.

## Conclusion

This study systematically elucidates the pivotal role of PRGs in the progression of EC through bioinformatics analysis. A prognostic risk model based on six characteristic genes was constructed, and its potential association with the tumor immune microenvironment was preliminarily revealed.

## Supplementary Information

Supplementary Material 1: Supplementary Figure S1. GO enrichment analysis of differential palmitoylation related genes. Supplementary Figure S2. Correlation analysis between characteristic genes and drug sensitivity.

Supplementary Material 2: Supplementary Table S1. Primers for reverse transcription quantitative polymerase chain reaction.

## Data Availability

The data and materials in the current study are available from the corresponding author on reasonable request.
