# Supplementary material for: Development and validation of palmitoylation-related genes characteristics for prediction of prognosis and immune landscape in endometrial cancer
Source: Hereditas. 2026 Jun 22;163:92. doi: 10.1186/s41065-026-00699-2 (PMC13540888; doi:10.1186/s41065-026-00699-2)
Supplement: Supplementary file 1 [file 41065_2026_699_MOESM1_ESM.docx]

**Development and validation of palmitoylation-related genes characteristics for prediction of prognosis and immune landscape in endometrial cancer**

**supplementary information:**

**Supplementary Figure S1. GO enrichment analysis of differential palmitoylation related genes.**

**Supplementary Figure S2. Correlation analysis between characteristic genes and drug sensitivity.**
